# Utilisation trends of rosiglitazone and pioglitazone in Australia before and after safety warnings

**DOI:** 10.1186/1472-6963-14-151

**Published:** 2014-04-04

**Authors:** Suvimol Niyomnaitham, Andrew Page, Adam La Caze, Karen Whitfield, Alesha J Smith

**Affiliations:** 1 School of Pharmacy, University of Queensland, 20 Cornwall Street, Woolloongabba, Australia; 2 Faculty of Medicine Siriraj Hospital, Mahidol University, Wanglang Road, 10700 Bangkok, Thailand; 3 School of Science and Health, Campbelltown campus, University of Western Sydney, Richmond NSW, Australia; 4 School of Pharmacy, University of Otago, Frederick Street, 9016 Dunedin, New Zealand

**Keywords:** Rosiglitazone, Pioglitazone, Safety warnings

## Abstract

**Background:**

A see on cardiovascular diseases and bladder cancer. The changes to the patterns of rosiglitazone and pioglitazone utilisation in Australia following the timing of these various health authority warnings such as the Australian Therapeutic Good Administration (TGA), European Medicines Agency (EMA) press releases or U.S. Food and Drug Administration (FDA) is unknown. This study investigated the utilisation patterns of rosiglitazone and pioglitazone in Australia before and after warnings of major drug authorities.

**Methods:**

We evaluated rosiglitazone and pioglitazone dispensing using the Pharmaceutical Benefit Scheme (PBS) subsidised drug dispensing data for the Australian population from February 2004 to July 2012. The World Health Organisation Anatomic Therapeutic Chemical (ATC)/Defined Daily Dose (DDD) system was used to compare the drug utilisation patterns following the announcements of EMA, FDA, and TGA safety warnings, which first occurred in May 2007. The DDD/1000population/day were examined in a series of time-series regression analysis with the drug safety warnings specified as interventions.

**Results:**

Rosiglitazone utilisation increased steadily from 2004 until reaching a peak at 1.96/1000population/day in January 2007. Then rosiglitazone use decreased significantly after the initial EMA press release and FDA warning on cardiovascular risk in May 2007 (with a 15.04% average monthly decline, p-value <0.001), however use did not significantly decrease after the TGA warning or subsequent EMA and FDA warnings. Pioglitazone utilisation proceeded rosiglitazone in September 2008 and remained above 1.5/1000/day during 2009–2010. However, pioglitazone utilisation has slightly declined after the FDA, EMA, and TGA warnings related to bladder cancer.

**Conclusions:**

Drug safety warnings were associated with a decrease in rosiglitazone and pioglitazone utilisation in Australia. Rosiglitazone began to decline prior to TGA warnings in December 2007, which suggests that Australian prescribers may have acted in response to scientific evidence or international safety warnings (EMA, FDA), prior to the response of the TGA. Minor effects were observed after bladder cancer warnings on pioglitazone utilisation.

## Background

Thiazolidinediones (TZDs) were approved for type 2 diabetes mellitus (DM) treatment based on efficacy studies, which showed a decrease in HbA1c, by 0.8-1.5% and improved insulin sensitivity [1,2]. Both TZDs, rosiglitazone and pioglitazone, were listed on the Australian Pharmaceutical Benefit Scheme (PBS) as subsidised second line therapy with either metformin or a sulfonylurea in November 2003 and later extended to triple oral therapy with metformin and a sulfonylurea and in combination with insulin [3].

In May 2007, a meta-analysis by Nissen and Wolski found a small increased risk in myocardial infarction and a borderline increase in cardiovascular death in patients treated with rosiglitazone [4]; however, another ongoing clinical trial evaluating cardiovascular outcomes of rosiglitazone showed cardiovascular events associated with rosiglitazone [5] to be inconclusive. Because cardiovascular disease can be a lethal complication in patients with diabetes mellitus, several studies have tried to establish the adverse cardiovascular effect associated with rosiglitazone treatment [6,7].

Since then, drug regulatory authorities have investigated these cardiovascular effects [8] and issued several warnings on the use of rosiglitazone [9,10]. The Australian regulatory authority, the Therapeutic Goods Administration (TGA) is responsible for ensuring the safety of medical products within Australia [11]. The TGA distributes safety information to healthcare professionals through the “Safety Advisory” on the TGA’s website, similar to that of the U.S. Food and Drug Administration (FDA) drug safety communication [12] and European Medicines Agency (EMA) press releases [13].

Since late 2008, PBS steadily limited the subsidisation of rosiglitazone use in combination with insulin and triple oral therapy [14]. On 1st July 2011, the PBS restricted prescription of rosiglitazone by requiring prior telephone approval [15].

While the meta-analysis raised a concern around the cardiovascular risk of rosiglitazone, a study of pioglitazone showed that in comparison it was a safe alternative with an insignificant increase in mortality, myocardial infarction and stroke [16]. Pioglitazone also reduced the risk of hospitalization for acute myocardial infarction in patients with type 2 diabetes in comparison with rosiglitazone [16,17]. In June 2011, a French study suggested an increased risk of bladder cancer in patients who were treated with pioglitazone for more than one year leading to a temporary withdrawal of pioglitazone by the French Agency [18]. Another study in the US also indicated a possible increase in bladder cancer risk in patients on pioglitazone for more than 2 years, compared with diabetes patients who were not receiving pioglitazone [19,20]. The TGA, as well as FDA and EMA, announced safety warnings outlining a possible risk of bladder cancer related to pioglitazone use in June-July 2011; however, there have been no further updates on this issue [21-23].

The increasing risk of cardiovascular disease with rosiglitazone led to a decrease in the utilisation patterns, in the US [24,25] and some countries in Europe [26,27]. It is expected that after the bladder cancer warnings, pioglitazone will follow a similar utilisation trend to that of rosiglitazone. However, it is plausible that pioglitazone use may have slightly changed as a result of prescribers weighing up the benefit in blood sugar control and prevention of cardiovascular events versus the possible increased risk of bladder cancer, which has a very low incidence (3 cases per 1000 pioglitazone users) [19,20]. The dispensing patterns of rosiglitazone and pioglitazone following the emerging cardiovascular event and safety warnings have not been described in Australia, although it is hypothesised that the trends will follow that of the US and Europe. This study aims to describe the patterns of rosiglitazone and pioglitazone use, and investigate the influential factors on changes of utilisation in Australia, with special focus on the safety warnings by TGA, FDA and EMA.

## Methods

### Data sources

Drug utilisation among populations over time can be examined using the World Health Organization Anatomic Therapeutic Chemical (ATC)/Defined Daily Dose (DDD) system [28]. Data on monthly dispensed medicines were obtained from the PBS database, a national administrative scheme which records drugs subsidised by the Government for Australian citizens. The PBS database captures all subsidized drug formulations, cost and amount of dispensing and period of drug dispensed by pharmacists for patients used at home [29]. Drug dispensed data on the PBS database were used in research studied and shown to represent trends of drug utilisation in Australia [30,31]. Rosiglitazone and pioglitazone are listed as subsidised drugs for all Australians therefore a complete record of dispensed medicines was obtained [3]. Denominator populations from Centrelink [32] and the Australian Bureau of Statistics [33] were used to calculate the DDD per 1000 population per day (the proportion of the population receiving a DDD of this drug per day). All the data for this study were aggregated, routinely collected data and publically available via government sources, therefore ethics approval was not required.

Australian drug safety warnings for rosiglitazone and pioglitazone were acquired from safety alerts and safety information for health professionals on the TGA website [34]. We accessed the EMA’s safety announcements, called “press releases” [35], and the FDA drug safety communication [36] from their official websites. Since mid-2007, major drug authorities have issued safety warnings related to rosiglitazone and pioglitazone. The first TGA announcement which highlighted the increased risk of ischemic heart disease associated with rosiglitazone was issued in December 2007 (TGA1) [37], followed by a second warning to avoid using rosiglitazone in patients with ischemic heart disease in September 2010 (TGA2) [38]. The FDA had three announcements related to cardiovascular risk of rosiglitazone [39]; firstly, a safety alert in May 2007 (FDA1) [40], a label update on heart-related risks in August 2007 (FDA2) [41], and then restrictions on rosiglitazone use in September 2010 (FDA3) [42]. There were four EMA press releases on risk of ischemic heart disease in May 2007 (EMA1) [43], October 2007 (EMA2) [44], January 2008 (EMA3) [45] and September 2010 (EMA4) [46]. While the TGA and FDA still allowed rosiglitazone on the market, the EMA suspended all medical products containing rosiglitazone across Europe in September 2010 [46].

For pioglitazone, the FDA issued a warning on a possible increased risk of bladder cancer in patients who used pioglitazone for longer than one year in June 2011 [23], followed by the same warnings in the EMA press release [21] and the TGA safety advisory [22] in July 2011.

Whilst there were other plausible types of information sent to prescribers with regards to the drug safety, it is recognized that the warnings from the FDA, EMA and TGA have a large influence on drug safety communication. For example, the pharmaceutical companies marketing these medicines did not implement changes to the Product Information until after the TGA announcement. In Australia, medical media picked up this side effect once it came out from the FDA as well as medical associations issued the FDA warning on their articles.

### Analyses

Monthly dispensing data of rosiglitazone and pioglitazone from January 2004 to July 2012 were converted to DDD/1000population/day. Descriptive trends in rosiglitazone and pioglitazone utilisation were examined in the time series of DDD/1000pop/day. The auto-regressive, integrated, moving average model (ARIMA) integrates the temporal size and direction dependency (autocorrelation) inherent in time-series data to better characterize changes in data over a period of time [47]. Autocorrelation functions (ACF) and partial autocorrelation functions (PACF) was used to obtain the best fitted model for analysis as well as the Bayesian Information Criteria. The percentage change in DDD/1000pop/day was used to remove the trend component of the time series before fitting into ARIMA models. The separate and combined effects of the announcement of the EMA, FDA, and TGA warnings on trends in rosiglitazone and pioglitazone utilisation were also investigated by fitting into ARIMA models. Impacts of drug safety warnings (interventions) on the subsequent observations were then investigated using the ARIMA model as a step-function (having a permanent and immediate impact on any subsequent trends). All statistical analyses were performed with a 5% statistical significance level using STATA 12.1 (StataCorp, College Station, TX).

## Results

**Figure 1:**
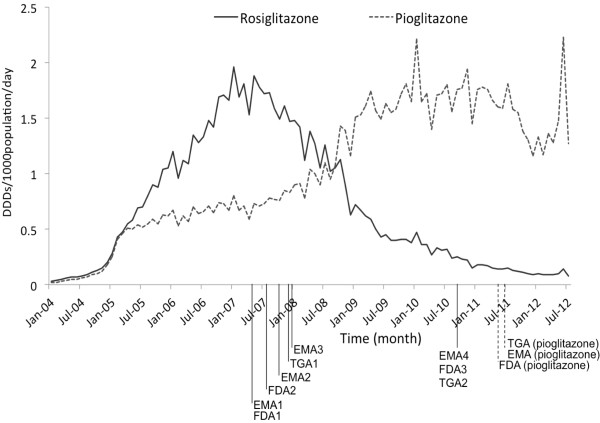
**Utilisation of rosiglitazone and pioglitazone by the Australian population between 2004–2012.** The drop-down lines indicate months of drug safety warnings issued. Notes: Rosiglitazone warnings: EMA1-Reminded the risk of rosiglitazone in patients with cardiac failure and other cardiac disorders including myocardial infarction. FDA1-Advised to evaluate the antidiabetic treatment options other than rosiglitazone in patients who have underlying heart disease and high risk of heart attack. FDA2-Adds box warnings for heart-related risks of rosiglitazone. EMA2-Suggested that rosiglitazone should only be used after careful evaluation of ischemic heart disease. TGA1-dvised that rosiglitazone should not be prescribed for patients with known ischemic heart disease or at high risk for ischemic heart disease. EMA3-Suggested that rosiglitazone must not be used in patients with an acute coronary disease. EMA4-Recommended suspension of all rosiglitazone-containing products. FDA3-Restricts access to rosiglitazone due to an elevated risk of cardiovascular events. TGA3-Reinforced that rosiglitazone should not be used in patients with known ischemic heart disease. Pioglitazone warnings: FDA-Announced the warnings on a possibly increased risk of bladder cancer in patients who used rosiglitazone for longer than one year. TGA-Advised the prescribers that use of pioglitazone for more than a year may be associated with an increased risk of bladder cancer. EMA-Recommends new contraindications and warnings for pioglitazone to reduce small increased risk of bladder cancer. TGA = Therapeutic Good Administration; EMA = European Medicines Agency; FDA = U.S. Food and Drug Administration.

A total of 1,686,087 rosiglitazone prescriptions and 2,405,881 pioglitazone prescriptions were dispensed during January 2004-July 2012. We calculated the monthly utilisation (DDD/1000population/day) using Australian population data, which was in the range of 20.1 million in 2004–22.9 million in 2012. As shown in Figure 1, the rosiglitazone utilisation increased steadily from 2004 and reached the peak in January 2007 with a defined daily dose of 1.96 per 1000 people per day. However, in May 2007, the trend of rosiglitazone utilisation started decreasing and remaining lower than 0.50 DDD/1000pop/day in May 2009 and 0.15 DDD/1000pop/day in July 2011. Pioglitazone utlisation has exceeded rosiglitazone use since September 2008 and remained stable during 2009–2010 (1.5-1.7 DDD/1000pop/day). Nevertheless, the trend of pioglitazone utilisation appeared to decrease in September 2011.

There are no seasonal autocorrelation detected for both rosiglitazone and pioglitazone utilisations. Based on visual inspection of PACF and ACF plots, an ARIMA (1,0,2) model best characterised for rosiglitazone data and pioglitazone data was best characterised as an ARIMA (1,0,1). Findings from ARIMA models indicated that the utilisation of rosiglitazone decreased significantly after the EMA1 and FDA1 warnings with −15.04% per month (p-value <0.001) (Table 1). Additionally, the utilisation of rosiglitazone also significantly decreased following warnings from FDA2, EMA2, TGA1, and EMA3. However, after adjustment for FDA2, EMA2, TGA1, and EMA3 for preceding warnings, effects were attenuated and were no longer statistically significant (Table 1). Later warnings relating to EMA4, FDA3, and TGA2 were not significantly associated with decreases in rosiglitazone use (Table 1).

**Table 1:** Effects of drug warnings on the utilisation of rosiglitazone and pioglitazone in Australia

**Drug authorities**	**Time**	**Warnings**	**Adjusted for**	**Coefficient**^**a**^	**95% CI**^**b**^	***p *** **value**
**Rosiglitazone: ARIMA (1,0,2) model**						
EMA1_FDA1	May 2007	Ischemic heart	-	−15.04	[−21.86, −8.22]	**<0.001**^**c**^
FDA2	Aug 2007	Label update heart related	EMA1_FDA1	−2.61	[−40.41, 35.20]	0.893
EMA2	Oct 2007	Ischemic heart	EMA1_FDA1, FDA2	1.94	[−95.49, 99.36]	0.969
TGA1	Dec 2007	Ischemic heart	EMA1_FDA1, FDA2, EMA2	−5.25	[−38.01, 27.51]	0.837
EMA3	Jan 2008	Ischemic heart	EMA1_FDA1, FDA2, EMA2, TGA1	−0.39	[−80.06, 79.28]	0.992
FDA3, TGA2, EMA4	Sep 2010	EU suspended,	EMA1_FDA1, FDA2, EMA2, TGA1, EMA3	1.25	[−8.99, 11.49]	0.811
		US restriction				
**Pioglitazone: ARIMA (1,0,1) model**						
FDA	June 2011	Bladder cancer	-	−5.76	[−13.91, 2.39]	0.166
EMA, TGA	July 2011	Bladder cancer	-	−6.57	[−14.80, 1.65]	0.117

^a^Coefficient = Percentage change in magnitude and direction after the intervention.

^b^CI = confidence interval.

^c^Statistical significance at *p* value <0.05.

TGA = Therapeutic Good Administration; EMA = European Medicines Agency; FDA = U.S. Food and Drug Administration; EU = European Union; US = United States of America.

For pioglitazone, although we can see a decline after the FDA, TGA, and EMA warnings on bladder cancer in June-July 2011, there is no statistically significant effect on subsequent pioglitazone use after fitting this into ARIMA model (Table 1).

## Discussion

The changes of rosiglitazone and pioglitazone utilisation were observed between 2004 and 2012 in Australia. It is always difficult to attribute cause to utilisation trends, however it is likely that increased marketing of TZDs may have contributed to the increasing trend of rosiglitazone during 2004–2006 or that fewer alternatives to metformin, sulfonylurea, and insulin were available at this time. Our results show a decreasing trend in rosiglitazone utilisation in the period after the drug authorities’ warnings in 2007–2008. Although the numbers of rosiglitazone prescriptions in Australia are relatively low in comparison to the UK, and North America, the overall trends are consistent with those shown in Europe and North America [24,26,27,48]. There are two possible explanations for the dip seen in April 2007. It might be a seasonal trend as the same fluctuation was noted in March-April 2006; however, this was not sensitive enough to be detected by the ARIMA model. Secondly, the dip is an artifact of the data, this is actually the utilisation on its way up which is demonstrated by the higher use again in May 2007.

The sharply decreasing utilisation trend is significantly attributable to the safety alert from meta-analysis study and the initial warnings from the EMA and FDA in May 2007. For the reason that the FDA issued the cardiovascular alert of rosiglitazone on the same day as publication by Nissen et al. [4], we could not distinguish the effects between the authority warnings and the publication. Furthermore, the effects of these warnings and associated literature are likely to be cumulative rather than a discrete effect on the following utilisation. Several restrictions in rosiglitazone subsidies from the PBS during October 2008-Febraury 2009 were also examined; however, these impacts are not significant after adjustment for previous warnings. As a result of the consecutive series of cardiovascular warnings on rosiglitazone since 2007 and the limited access on PBS, the numbers of rosiglitazone prescriptions have remained lower than 5,000 per month since 2010.

Australian utilisation of pioglitazone was less than half of rosiglitazone during 2005–2007 and the increasing trend in use was moderate compared to the Netherlands and the US [25,27]. From 2008–2010, when peak levels were reached, the increase in pioglitazone nearly mirrors the decline in rosiglitazone. The findings suggest that prescribers might have replaced rosiglitazone with the same drug class pioglitazone [24,49], due to the reported cardiovascular benefits of pioglitazone, and no clinical outcome associated with an increase risk of ischemic heart disease that was seen with rosiglitazone [7,17]. While the decreasing trend of pioglitazone was observed in the US and Europe in 2008 [27,49], Australian pioglitazone utilisation plateaued until 2011. The delay in decreasing trend compared to that of other countries may be attribute to limited availability of second-line and third-line therapy alternatives such as sitagliptin (was not PBS subsidised until August 2008) or exenatide (was not PBS subsidised until August 2010) [50]. The US and UK data [49,51] show that the number of other new drugs, which were available in their markets since 2007 such as sitagliptin and exenatide, increased after the cardiovascular alerts of TZD. Nevertheless, Figure 1 shows the decline in the utilisation of pioglitazone after July 2011. This decreasing trend may have been caused by more alternative treatments on the PBS or the safety concern of increased risk of bladder cancer in long-term users of pioglitazone. Although, this decline was of a lesser magnitude than for rosiglitazone, prescribers may consider the risk/benefit ratio, where the benefits of pioglitazone in lowering blood sugar outweigh the possible risk of bladder cancer [52]. However, more data points following this bladder cancer risk might be needed to examine the true effect of this warning.

Since TGA safety warnings are considered by the Australian Department of Health and Aging to be first-line alerts to Australian prescribers, we would expect to see a significant effect on these utilisations. However, the fact that a) the decline in rosiglitazone use occurred prior to the first TGA warning, and b) after we adjusted for the preceding EMA, FDA warnings, we could not see a significant effect of the TGA warning on utilisation trends suggests that Australian prescribers were aware of the international warnings as well as the safety information from the literature. This might be associated with the way that information was delivered, since Australian warnings were delayed, less frequently communicated, and accessed compared to the FDA and European warnings [38,53]. Australian prescribers may receive safety information from medical articles or media that referred to the US or European warnings. A further qualitative study is being conduct to gain the insight into sources of drug safety information among Australian prescribers.

Since time series model prediction is based on the pattern of drug use in the past confounding influences on data may be difficult to disentangle. Although trends can be impacted by temporal changes in drug supply or the way data are recorded, we did not find those problems during study period. Furthermore, the Australia PBS data is aggregated data collected for administrative purposes, which does not link utilisation to the prescribing data in clinical settings. Therefore, clinical reasons for the decrease in dispensing cannot be fully investigated, nor primary non-compliance in patients be established.

The strength of this study is that it captures almost all prescriptions dispensed over 2004–2012 in total Australian population (private prescriptions represent a very small percentage of all prescriptions). This is achieved because rosiglitazone and pioglitazone are ‘high’ cost drugs that are government subsidised in Australia. This allowed us to investigate the patterns of population based thiazolidinedione utilisation.

## Conclusions

The utilisation of rosiglitazone significantly decreased following the authorities’ safety warnings on ischemic heart disease. The pattern of rosiglitazone utilisation started declining significantly prior to the TGA warning in December 2007; therefore it appears that Australian prescribers were alerted by the literature and international warnings such as EMA and FDA. In contrast, pioglitazone utilisation increased during the rosiglitazone warning period during 2007–2010. In comparison to the US and Europe, the decline in pioglitazone trend was much more deferred due to no available second and third line therapies in Australia. Despite concerns surrounding the possible risk of bladder cancer with long term use of pioglitazone, this study showed weaker effects of safety warnings on bladder cancer and pioglitazone utilisation. A number of publications have studied the effect of authorities’ warnings in the US and Europe to improve their warning systems [27,54,55]. This is one of the first studies to date that has investigated utilisation patterns in relation to drug safety warnings in Australia and suggests that TGA warnings may not affect prescribing in cases such as this where prescribers may be attuned to particular medicine safety issues described in earlier international warnings or literature. Further research is needed to understand how and when prescribers obtain drug safety information in Australia. This is particularly pertinent as Australia and New Zealand look to combine their drug safety warning systems.

## Abbreviations

TGA: Therapeutic good administration; EMA: European medicines agency; FDA: U.S. food and drug administration; PBS: Pharmaceutical benefit scheme; DDD: Defined daily dose; TZD: Thiazolidinedione; DM: Diabetes mellitus; ARIMA: Auto-regressive, integrated, moving average model; ACF: Autocorrelation functions; PACF: Partial autocorrelation functions; CI: Confidence interval.

## Competing interests

No sources of funding were involved in this study. The authors declare that they have no competing interests.

## Authors’ contributions

SN, AC, KW, and AS were responsible for developing the study and method. SN and AP participated in the data collection, statistical analysis and result interpretation. All authors contributed to manuscript writing and all revisions of the manuscript. All authors read and approved the final manuscript.

## Pre-publication history

The pre-publication history for this paper can be accessed here:

http://www.biomedcentral.com/1472-6963/14/151/prepub
